# Effects of dietary lipid level and environmental temperature on lipid metabolism in the intestine and liver, and choline requirement in Atlantic salmon (*Salmo salar* L) parr

**DOI:** 10.1017/jns.2023.45

**Published:** 2023-05-25

**Authors:** Daphne Siciliani, Trond M. Kortner, Gerd M. Berge, Anne Kristine Hansen, Åshild Krogdahl

**Affiliations:** 1 Department of Paraclinical Sciences, Norwegian University of Life Sciences, Ås, Norway; 2 NOFIMA, Sunndalsøra, Norway; 3 Biomar AS, Havnegata 9, Trondheim 7010, Norway

**Keywords:** Choline requirement, Fish nutrition, Gut health, Lipid accumulation, Plant feed

## Abstract

Choline was recently established as an essential nutrient for Atlantic salmon at all life stages. Choline deficiency is manifested as an excessive accumulation of dietary fat within the intestinal enterocytes, a condition known as steatosis. Most of today's plant-based salmon feeds will be choline-deficient unless choline is supplemented. Choline's role in lipid transport suggests that choline requirement may depend on factors such as dietary lipid level and environmental temperature. The present study was therefore conducted to investigate whether lipid level and water temperature can affect steatosis symptoms, and thereby choline requirement in Atlantic salmon. Four choline-deficient plant-based diets were formulated differing in lipid level of 16, 20, 25 and 28 % and fed to salmon of 25 g initial weight in duplicate tanks per diet at two different environmental temperatures: 8 and 15 °C. After 8 weeks of feeding, samples of blood, tissue and gut content from six fish per tank were collected, for analyses of histomorphological, biochemical and molecular biomarkers of steatosis and choline requirement. Increasing lipid level did not affect growth rate but increased relative weight and lipid content of the pyloric caeca and histological symptoms of intestinal steatosis and decreased fish yield. Elevation of the water temperature from 8 to 15 °C, increased growth rate, relative weight of the pyloric caeca, and the histological symptoms of steatosis seemed to become more severe. We conclude that dietary lipid level, as well as environmental temperature, affect choline requirement to a magnitude of importance for fish biology and health, and for fish yield.

## Introduction

Increasing use of plant ingredients in diets for Atlantic salmon, which inevitably changed content of micronutrients as well as antinutrients, have been suggested to be a possible cause of increased gut health challenges observed in farmed Atlantic salmon^(1,2)^. Steatosis in the pyloric caeca is one of the frequent symptoms^(3)^, characterised by a whitish and swollen appearance of the intestine and by the presence of lipid droplets accumulating in the enterocytes. In severe cases, the condition is known as lipid malabsorption syndrome (LMS), and lipidic digesta is present throughout the intestinal tract, with lipid loss and pollution of the environment as concequenses^(4)^. The steatosis may be the result of various suboptimal conditions in the complex process of lipid digestion and absorption. Deficient supply of long-chain fatty acids may alter fatty acid metabolism in enterocytes and result in intracellular lipid accumulation, as shown by Bou *et al.*^(5)^. The latter study also showed that the fatty acid composition of the diet, intestinal mucosa, and liver differed, indicative of fatty acid metabolism in both body compartments. A study of intracellular trafficking of fatty acids employing the rainbow trout enterocyte cell line RTgutGC, showed differences between fatty acids regarding the accumulation of lipid droplets in the cytosol, being higher for oleic acid than palmitic acid^(6)^. A study conducted by Bogevik *et al.*^(7)^ on enterocytes isolated from Atlantic salmon highlighted that elongation and desaturation of fatty acids is limited in these cells. It is, therefore, possible that variation in dietary fatty acid composition might affect lipid metabolism and turnover, and thereby the degree of lipid accumulation in the pyloric caeca. Additionally, it has been observed by Ballester-Lozano *et al.*^(8)^, that gilthead sea bream (*Sparus aurata*) fed to a diet poor in *n*-3 LC-PUFA showed signs of lipid accumulation in the proximal intestine. However, the more likely explanation for the excessive lipid accumulation in enterocytes is deficient supply of choline, which will limit the capacity for production of lipoproteins for export of fat from enterocytes to the internal compartments of the fish^(9,10)^. In a series of studies in Atlantic salmon, an inverse relationship between symptoms of steatosis and dietary choline level has been demonstrated^(9,11,12)^. Choline is a water-soluble organic compound involved in a broad range of critical physiological mechanisms across all life cycle stages. Among its several functions choline plays a pivotal role in forming the hydrophilic head of the phosphatidylcholine molecule, a major component of the very low-density lipoprotein (VLDL) complex^(13)^, which carries triglycerides synthesised in the intestine and liver to adipose tissue, muscles and other organs. Several signs of choline deficiency, such as high hepatic lipid concentration, reduced growth performance, early death and poor feed efficiency, have been reported in fish species such as carp (*Cyprinus carpio*)^(14)^, lake trout (*Salvelinus namaycush*)^(15)^, rainbow trout (*Oncorhynchus mykiss*)^(16)^, yellow perch (*Perca flavescens*)^(17)^, channel catfish (*Ictalurus punctatus*)^(18)^, cobia (*Rachycentron canadum*)^(19)^ and yellowtail kingfish (*Seriola lalandi*)^(20)^. However, only a few other studies with Japanese eel (*Anguilla japonica*)^(21)^ and faba grass carp (*Ctenopharyngodon Idella*)^(22)^ have addressed the lipid transport capacity in the intestine and described the characteristic whitish appearance of the intestine, as a result of a deficiency of choline.

The National Research Council^(23)^ has recognised choline as an essential nutrient for several fish species, but for some, including the Atlantic salmon, the conclusion has so far been that choline is essential only at the very young stages. Choline is present in numerous raw materials, but the content differs greatly, in particular between marine and plant ingredients. Therefore, supplementation with choline is necessary for many fish species fed diets based on plant ingredients. Choline requirement of fish seems to vary between species and between experiments within species. The review of Mai *et al.*^(24)^ reports estimates of choline requirement ranging from 500 mg/kg diet in a study of hybrid striped bass (*Morone saxatilis _ Morone chrysops*), to 4000 mg/kg diet^(23)^ in a rainbow trout study. However, estimates of requirement in rainbow trout differ greatly between studies. One indicates a requirement of 800 mg/kg and suggests that many factors, biotic as well as abiotic, and not at least choice of deficiency biomarker, may be of importance for the requirement. Regarding Atlantic salmon, only one published study, by Hansen *et al.*^(10)^, addresses choline requirement at seawater stages. The results showed that choline is essential for salmon also for fish in seawater. The obtained data made Hansen *et al.*^(10)^ suggest that a dietary choline inclusion of 3350 mg/kg is necessary in order to avoid choline deficiency signs in the pyloric caeca of post-smolt Atlantic salmon^(10)^ weighing around 450 g and fed a diet with 29 % lipid. The results call for further studies to define requirement under conditions which might be more demanding than those employed in the study. In most of previous studies of choline requirement in fish, the main biomarkers have been weight gain and liver lipid content^(18,25–28)^. However, in Hansen *et al.*'s studies, growth, liver index and lipid content were rather insensitive markers of variation in choline supply. The characteristics of the mucosa of the pyloric intestine (PI), on the other hand, such as organosomatic index, level of enterocyte vacuolation and expression of several genes involved in lipid assembly in the enterocytes, storage and transport were among the response variables which showed a clear dose-response relationship with dietary choline inclusion.

The experiment described in the present report is the first of two experiments following up the study of Hansen *et al.*^(10)^. These studies aim to identify conditions which might affect choline requirement in Atlantic salmon importantly. Choline's role in lipid transport suggests that dietary lipid level, lipid quality, feed intake, size, developmental stage, environmental temperature and day length may affect the requirement, and that such conditions interact in their effects on choline requirement, as further elaborated on in the discussion. The conditions selected for investigation in the present experiment were dietary lipid level and environmental temperature. The following addresses effects of lipid quality and fish size. The results of the present experiment, will, together with a second serve as basis for planning a final dose-response study with choline fed to the fish under the most choline-demanding conditions.

## Materials and methods

To limit the use of fish for welfare reasons, and of experimental and laboratory facilities for cost and time reasons, a screening strategy was chosen for the experiments, i.e. observing effects in fish fed choline-deficient diets, containing choline in a range sensitive for variation in choline supply, i.e. less than 50 % of the level indicated as sufficient in the study of Hansen *et al.*^(10)^. To get an indication of the quantitative aspects of the observed variation, the observed variation in the biomarkers were compared to the corresponding variation in biomarkers in Hansen *et al.*'s study induced by variation in choline level and taken as indication of change in choline requirement. In Hansen *et al.*'s study choline was supplemented as choline chloride, a condition which might raise questions regarding variation in digestibility between sources. In a recent study, choline in plant-based diets, without and with choline chloride supplementation, showed digestibility above 90 % for all diets^(29)^, highest for the supplemented diets. As the condition in the present and previous experiment differed to some extent, the indicated change in choline requirement should be considered only indicative, and not to represent an exact estimate of effects on the requirement.

### Diets

Four similar diets with high content of plant ingredients, deficient choline level, and varying in lipid level from 16, 20, 25 and 28 % were formulated. The diets were formulated to be iso-nitrogenous, as often recommended for nutritional studies. This means that with increasing lipid content in the diet, ingredients with low protein content had to be replaced with ingredients with a higher protein level. For each step up of lipid in the diet, the alteration in these plant ingredients were changed proportionally, i.e. increasing level of maize gluten (80 % protein) and decreasing a mixture of SPC and wheat, all of high quality. The variations were expected not to change nutrient digestibility or passage rate. The formulations and macronutrient compositions are shown in Tables 1a and 1b. The diets were supplemented with standard vitamin and mineral premixes in accordance with NRC guidelines (2011)^(23)^. Yttrium oxide (0⋅50 g/kg) was added as an inert marker for estimation of apparent nutrient digestibility. The diets were formulated to contain level of long-chain ω3 fatty acids well above requirements to avoid other causes of steatosis than choline deficiency. The experimental diets were produced by extrusion (feed pellet size 6 mm) using a BC 45 twin screw extruder (Clextral, France). Upon arrival of the diets at the experimental site, fat leakage from the diet with 28 % fat was discovered. A new diet was made with higher content of soy lecithin for better emulsification. The consequences of the intervention, i.e. a dietary choline level higher than planned, was not realised until after the feeding period was completed. However, this unexpected event was found not to significantly disturb the aims of the experiment. Even though the results obtained for the diets containing 16, 20 and 25 % of lipid level were sufficient to draw conclusions regarding the aims of the study, the results for the 28 % diets are included in this presentation. In fact, the higher choline level in the 28 % lipid diet supplied useful additional information, confirming not only that the severity of the steatosis in the pyloric caeca varies with choline supply, but also the important role of choline for sufficient lipid transport and metabolism in the pyloric caeca. Additionally, to include the results for the diet with 28 % lipid, also strengthens the statistical power of the experiment.

**Table 1a.** Diet receipts and results and nutrient content as formulated and analysed

	Diets			
	16	20	25	28
*Ingredients, %*				
Fish meal NA Con-Kix 72 %	20⋅0	20⋅0	20⋅0	20⋅0
Soya SPC >62 %, non gmo	24⋅2	16⋅1	8⋅1	0⋅0
Wheat gluten 80	0⋅0	5⋅8	11⋅6	17⋅4
Maize gluten 60 (min. 58 %)	10⋅0	9⋅6	9⋅3	8⋅9
Pea protein 65 %	13⋅3	13⋅4	13⋅6	13⋅7
Wheat (Milling quality)	14⋅8	11⋅6	8⋅4	5⋅2
Tapioca starch	0⋅0	0⋅7	1⋅3	2⋅0
Fish oil	3⋅2	4⋅9	6⋅7	8⋅5
Rapeseed oil, crude	7⋅4	10⋅3	13⋅3	14⋅9
Premix	0⋅5	0⋅5	1⋅1	1⋅1
Lecithin (Soy)	0⋅8	0⋅8	0⋅8	2⋅0
*Formulated nutrient composition*				
Formulated lipid (%)	16⋅0	21⋅0	26⋅0	31⋅0
Crude protein (%)	42⋅0	42⋅0	42⋅0	42⋅0
Gross energy (MJ/kg)	17⋅3	18⋅8	20⋅5	22⋅3
Total choline (mg/kg)	1619	1590	1560	1542
*Analysed nutrients*				
Dry matter (%)	93⋅0	92⋅3	92⋅0	92⋅9
Crude protein (%)	47⋅9	46⋅0	44⋅3	44⋅7
Lipid (%)	15⋅5	20⋅4	25⋅1	27⋅6
Choline (mg/kg)*	1930	1830	1940	2310
Nitrogen free extracts (%)	22⋅4	19⋅8	15⋅4	13⋅4
Ash (%)	8⋅6	7⋅4	6⋅8	7⋅5
Energy (MJ/kg)	20⋅6	22⋅3	23⋅4	24⋅0
Yttrium (%)	0⋅00044	0⋅00056	0⋅00049	0⋅00103

* See materials and methods for explanation and considerations regarding the high choline level in the D28 diet.

**Table 1b.** Content of fatty acids in the diets, % of sum of fatty acids*

Fatty acid	Diets			
	16	20	25	28
C14:0	2⋅05	2⋅10	2⋅05	2⋅12
C16:0	9⋅81	9⋅54	9⋅15	9⋅58
C16:1*n*7	2⋅76	2⋅98	2⋅97	3⋅03
C18:0	2⋅14	2⋅15	2⋅89	3⋅01
C18:1*n*9c	31⋅1	31⋅9	33⋅5	33⋅5
C18:2*n*6c	19⋅3	18⋅5	18⋅4	18⋅4
C20:0	0⋅36	0⋅37	0⋅50	0⋅48
C20:1*n*11	3⋅02	3⋅14	3⋅16	2⋅94
C20:2*n*6	0⋅16	0⋅17	0⋅18	0⋅18
C20:3*n*6	0⋅03	0⋅03	0⋅03	0⋅03
C20:4*n*6	0⋅15	0⋅15	0⋅14	0⋅17
C22:1*n*11	3⋅45	3⋅76	3⋅79	3⋅40
C18:3*n*3	5⋅20	5⋅18	5⋅16	5⋅21
C20:5*n*3	3⋅42	3⋅42	3⋅22	3⋅17
C22:5*n*3	0⋅22	0⋅22	0⋅21	0⋅22
C22:6*n*3	3⋅64	3⋅65	3⋅43	3⋅34
∑ Identified acids	88⋅0	88⋅7	90⋅8	90⋅9
∑ *n*-3	12⋅5	12⋅5	12⋅1	12⋅0
∑ *n*-6	19⋅8	18⋅9	18⋅8	18⋅9
∑ MUFA	40⋅6	42⋅2	43⋅7	43⋅2
∑ Saturated	15⋅1	15⋅0	16⋅2	16⋅8

MUFA,  monousaturated fatty acids.

* The results show the area of peak for the fatty acid in the chromatogram given as % of sum of the fatty acids areas of which some were not identified.

### Experimental animals and conditions

The feeding trial was conducted at Nofima's Research Station in Sunndalsøra (NO), which is approved by Norwegian Animal Research Authority (NARA) and operates in accordance with Norwegian Regulations of 17th of June 2008 No. 822: Regulations relating to Operation of Aquaculture Establishments (Aquaculture Operation Regulations). Trial fish were treated in accordance with the Aquaculture Operation Regulations during the experiment. As no harmful procedures were forced upon the fish before euthanisation, a specific permission was not needed for this experiment. Atlantic salmon with an average initial weight of 24 g were assigned to 0⋅6 × 0⋅6 m^2^ (125 L) flow through tanks, 140 fish per tank. Each diet was fed to fish in duplicate tanks for each dietary treatment, and each water temperature: 8 and 15 °C, i.e. a total of sixteen tanks. The temperature range is considered to cover the optimal range for Atlantic salmon^(30)^. A 24 h light regime was employed. Water temperature was measured daily, and dissolved oxygen weekly to secure 80–100 % of saturation. To secure feeding to satiation, the fish were fed 15 % in surplus of anticipated requirement, according to feeding tables and expected growth rate, using belt feeders.

### Sampling

After 5 weeks of feeding, the biomass in each tank was reduced to keep biomass at a lower level to ensure sufficient oxygen supply. A total of fifty fish were randomly removed from each tank at the highest temperature, and twenty fish per tank at the lower temperature, to similar biomasses in all tanks. After another 3 weeks of feeding, six fish in fed state from each tank were sampled randomly, anaesthetised with tricaine methane-sulfonate (MS-222) and killed by a sharp blow to the head, in accordance with the Norwegian Animal Welfare act. Weight and length of each sampled fish were recorded. Fish remaining in the tanks at the end of the sampling were weighed in bulk. Total weight gain was calculated by adding up the weights of the sampled and the remaining fish. Blood was sampled from the caudal vein into vacutainers with lithium heparin and kept on ice. After centrifugation, plasma was collected in 2 ml aliquots, frozen in liquid nitrogen and kept at 80 °C. Following blood sampling, the fish were opened ventrally, and the abdominal organ package was removed from the abdominal cavity. The liver was separated from the package and weighed. Thereafter, the intestine, freed of external fat was sectioned as follows: PI, the section from the pyloric sphincter to the most distal pyloric caecum; mid intestine (MI), from the latter pyloric caecum to the earliest area with higher diameter and darker pigmentation, distal intestine (DI), from the latter end of the MI to the anus. The sections were opened and digesta from the section were collected, snap-frozen in liquid N_2_ and stored at −80 °C. Those fish found with empty intestine were excluded from the sampling. Each intestinal section was then weighed before tissue samples were collected for histological and gene expression analyses. The samples for histological examination were immediately fixed in 10 % neutral-buffered formalin (4 % formaldehyde) and kept at room temperature. Whereas the samples for gene expression analyses were rinsed in sterile saline water, stored in RNA later® at 4 °C, and moved to −40 °C after 24 h. The fish that were removed for reduction of biomass at 5 weeks were stripped for faeces, while the remaining fish in the tanks were stripped for faeces at termination of the experiment, method as described by Austreng^(31)^. The faecal samples were pooled for each tanks, frozen in liquid N_2_ and stored at −20 °C.

### Chemical analyses of feed, gut contents, plasma, PI and liver including fatty acid analyses (FAME)

Samples of the feed and faeces were analysed for dry matter (DM), ash, crude protein (CP) and crude fat (CF) and energy at Nofima, Sunndalsøra. Fatty acid content was analysed at the Department of Animal and Aquacultural Sciences, Norwegian University of Life Sciences, Ås, Norway. DM was determined by drying the samples to a constant weight at 103 °C. Determination of ash content, samples were combusted at 550 °C for 10 h. Total nitrogen was analysed by Kjeldahl auto analyzer, and energy by bomb calorimetry (Parr 1271 adiabatic bomb calorimeter). Fatty acid composition was quantified by the FAME method described by O'fallon^(32)^. Choline level in the diets was analysed by Eurofins. The method involves extraction by methanol and water, alkaline hydrolysis to free choline from phosphatidyl choline, and quantification by isotope dilution LC-MSMS. The choline results show total choline, free and bound.

Analyses of yttrium content in feed and faeces were carried out by pre-digestion with concentrated ultrapure HNO_3_ at 250 °C using a Milestone microwave UltraClave III (Milestone Srl, Sorisole, Italy). Samples were then diluted (to 10 % HNO_3_ concentration), and yttrium was determined by inductively coupled plasma optical emission spectrometry (ICP-OES analysis) with a PerkinElmer Optima 5300 DV (PerkinElmer Inc., Shelton, CT, USA). The plasma nutrient and cholesterol analyses were carried out at the Central Lab of the Faculty of Veterinary Medicine, by standard hospital methods.

### Histology

**Figure 1.**
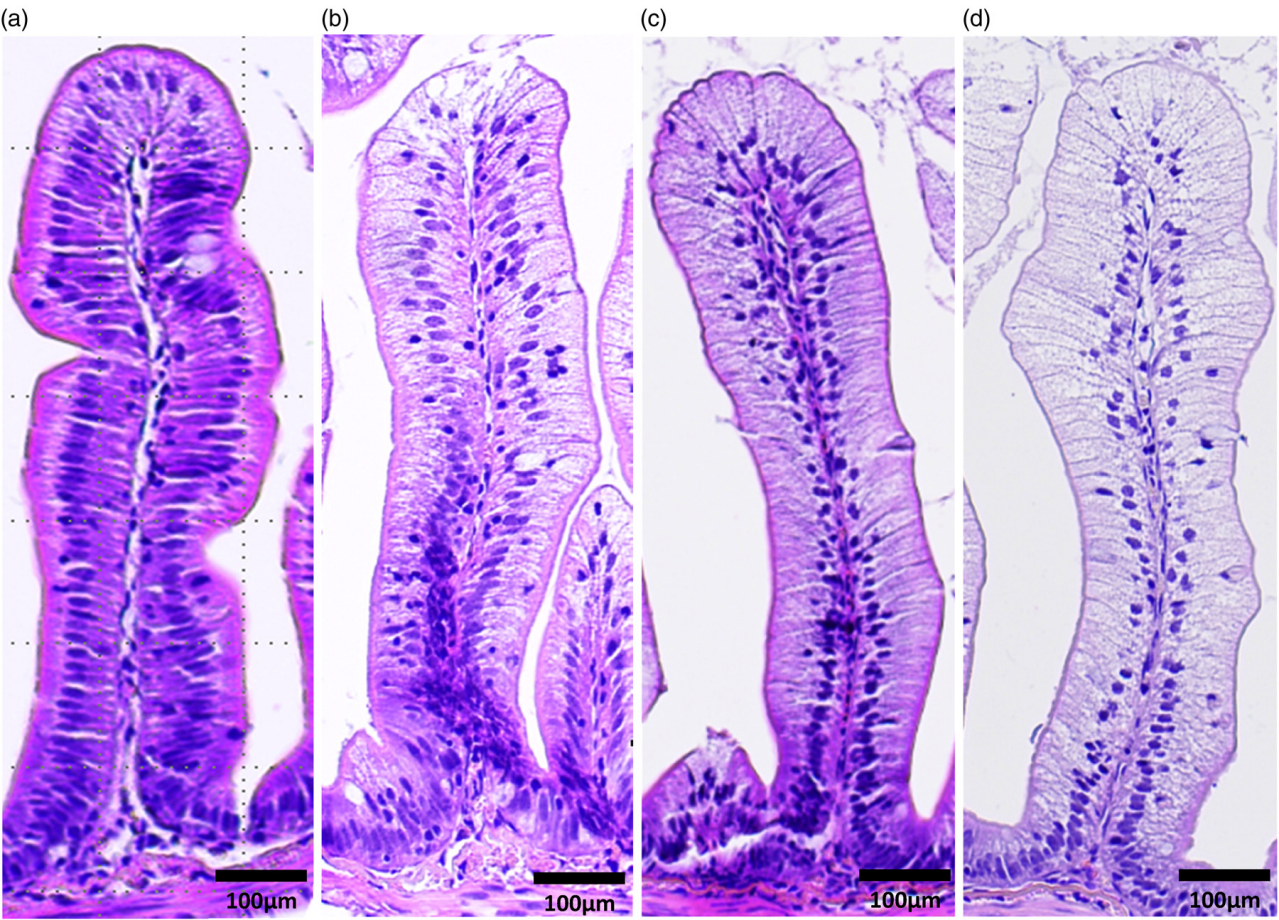
Histological severity of vacuolation of the pyloric caeca tissue (steatosis) representative for (a) normal, (b) moderate, (c) marked and (d) severe.

Pyloric caeca samples were processed at the Norwegian University of Life Sciences (NMBU) using standard histological techniques: dehydration in graded ethanol, clarification in xylene, embedding in paraffin and sectioning of 5 μm thick sections. The sections were then dewaxed, re-hydrated and stained with haematoxylin and eosin to perform the histological evaluation. The samples were randomised, and the main signs of vacuolation were assessed using a light microscope. According to the proportion of tissue affected by the presence of lipid-like vacuoles, swollen and irregular cells and condensed nuclei, the severity of the syndrome was scored as Normal (≤10 %), Mild (10–25 %), Moderate (25–50 %), Marked (≥50 %) and Severe (≥75 %) (Fig. 1).

### RNA extraction, cDNA synthesis and gene expression analyses

Gene expression analysis was performed from ninety-six samples collected from the pyloric caeca of the same six fish per tank used for the preceding analyses. Gene expression profiling was conducted by Quantitative Real-Time PCR (qPCR) following the MIQE guidelines^(33)^. Total RNA was extracted from pyloric caeca samples (around 30 mg) using a Ultraturrax homogenizer, TRIzol® reagent (Invitrogen, ThermoFisher Scientific) and chloroform according to the manufacturer's protocol. Obtained RNA was DNase treated (TURBO™, Ambion, ThermoFisher Scientific) and purified with PureLink RNA mini kit (Invitrogen, ThermoFisher Scientific). RNA purity and yield was measured using a NanoDrop ND-1000 Spectrophotometer (NanoDrop Technologies) and RNA integrity was assessed by 2100 Bioanalyzer by the use of a RNA Nano Chip (Agilent Technologies). Total RNA was stored at −80 °C for upcoming analyses. Before proceeding with the synthesis of the first-strand cDNA, the RNA from the fish of each tank was pooled two by two. Afterward, 0⋅8 μg of the total pooled RNA, oligo (dT)_20_ primers and Superscript III in 20 μl reactions (Invitrogen) were used to conduct the synthesis. To achieve negative controls, the same process was performed, omitting RNA and enzyme. cDNA was then diluted 1:10 and stored at −20 °C before the following qPCR procedure. The gene expression profiling was performed on a pool target genes involved in lipid metabolism, lipid uptake and transport and phosphatidylcholine synthesis. The primers to be used in the qPCR reaction were obtained from literature and from previous works conducted in this group^(9,12)^. Additional information concerning genes name or primers source, efficiency and size, is shown in Supplementary Table S1. For new assays, primer optimisation was carried out by PCR gradient assays, followed by assessment of PCR reaction efficiency (E) using serial dilutions of a pool of randomly selected cDNA samples. A LightCycler 96 (Roche Diagnostic) was used to perform DNA amplification and gene expression analyses. Each reaction mix contained 2 μl PCR-graded water, 5 μl of LightCycler 480 SYBR Green I Master mix (Roche Diagnostics) and 0⋅5 μl of both forward and reverse primer. Every sample was analysed in duplicate alongside a no template control. The three-step qPCR program included an enzyme activation step at 95 degrees for 5 min, followed by 40–45 cycles of 95 degrees (10 s), 55, 58, 60 or 63 °C (10 s), and 72 °C (15 s). Quantification cycle (Cq) values were calculated using the second derivative method. The products obtained from the qPCR were assessed analysing the melting curve. To perform relative normalisation of the qPCR assay, a pool of reference genes was selected as suggested by Kortner *et al.*^(34)^. Considering the absence of a universally stable reference gene, we tested the stability of a selected pool already used by our group for several previous studies conducted on the pyloric caeca of Atlantic salmon^(9,11)^. The most stable reference genes, selected based on their overall coefficient of variation (CV) and their interspecific variance were RNA polymerase II (*rnapoli*), hypoxanthine phosphoribosyl transferase 1 (*hprt1*) and glyceraldehyde-3-phosphate dehydrogenase (*gapdh*). Mean normalised expression of the target genes was calculated from raw Cq values by relative quantification^(35)^. The panel of target genes was the same used by Hansen *et al.*^(8–10)^ and it is represented by a set of genes involved in lipoprotein assembly, choline and phosphatidylcholine synthesis and cholesterol synthesis. As also done in the previously cited studies by Hansen *et al.*, among the whole pool of genes, four were selected as main molecular biomarkers because of their particular receptiveness to steatosis: *plin2*, *apoA-I*, *apoA-IV* and *pcyt1α*.

### Calculations

Fish growth was calculated as specific growth rate (SGR, percentage growth per day)= ((ln FBWg/ln IBWg)/D) × 100. IBW and FBW represent the initial and final body weight as tank means, and D represents the number of feeding days. The condition factor was calculated as: CF =((FBW × 100)/fork length cm^3^). The organosomatic indices (OSI) were calculated as: (organ weight g/body weight g) × 100. Apparent digestibility (AD) for each nutrient was determined by using yttrium oxide (Y_2_O_3_) as inactive marker and estimated as follows: AD*_n_*=100 – (100 × (*M*_feed/_*M*_faeces_) × (*N*_feed/_*N*_faeces_)), where *M* represents the percentage of Yttrium oxide in feed and faeces and *N* represents the percentage of a specific nutrient in feed and faeces.

### Statistical analyses

In light of the fact that this experiment was a screening study and had a regression design with close relationship between the five treatments, duplicate observations were considered sufficient. The design gives 11 degrees of freedom for the error term, and a reasonably accurate estimate of the error. Tank mean was used as the statistical unit. The results were subject to two-way analyses of variance (ANOVA) with lipid level and temperature as class variables. The Shapiro–Wilk test was used to assess the normality of the variance. The Tukey's test was used rank treatments in the molecular analyses, while Duncan's test was used for the other results. The level of significance for all analyses was set at *P* < 0⋅05, and *P*-values between 0⋅05 and 0⋅1 were considered to indicate a trend in effects as indicated in the text. The plan was to evaluate the results by regression analyses, but the event described above in the production of the D28 diet, made the result for the diet with the highest lipid content unsuitable for regression analyses. The two-way ANOVA was therefore chosen as the most suitable method.

## Results

### Organosomatic indices (OSI), histological evaluation and lipid content in pyloric and liver tissue

**Figure 2.**
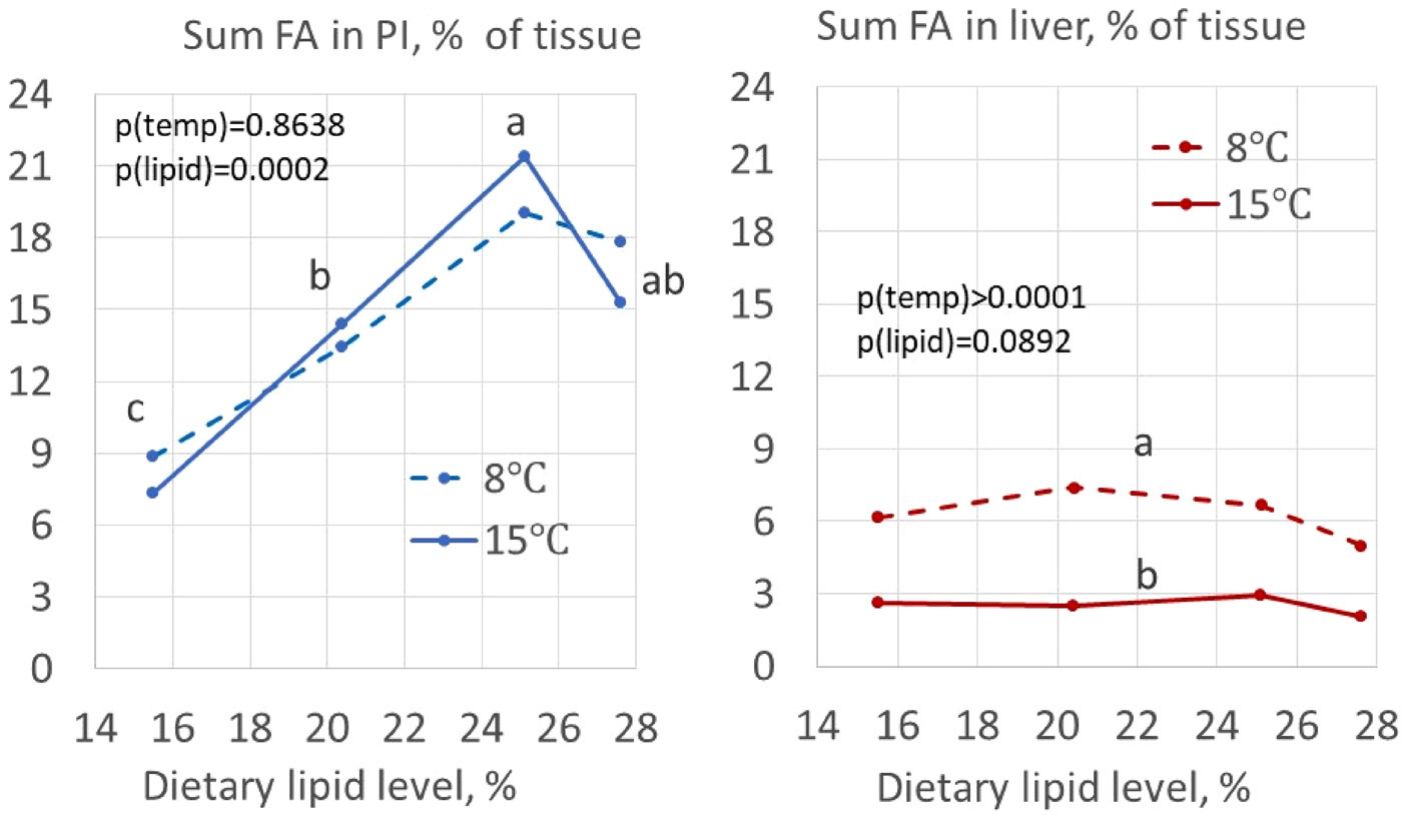
Effects of dietary lipid level on sum of fatty acids (Sum FA) in tissue from pyloric intestinal (left, PI) and liver (right).

**Figure 3.**
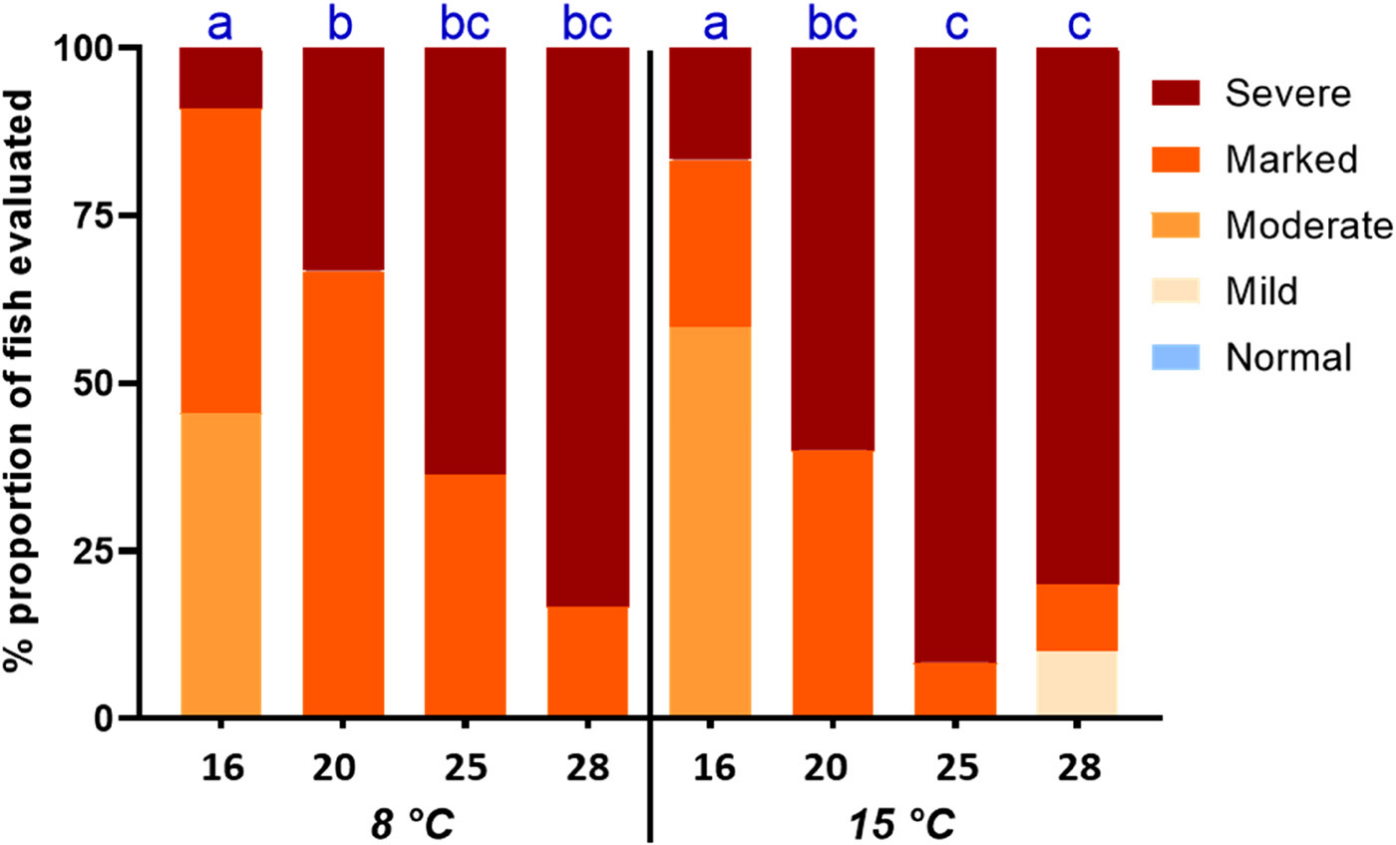
Number of pyloric caeca tissue scored for enterocyte steatosis. *X*-axis presents dietary lipid level at two rearing water temperatures of 8 and 15 °C. Superscript letters represent a significant statistical difference.

Relative organ weight was calculated for PI (OSIPI) and liver (HSI) (see Table 2). Increasing fat level in the diet increased OSIPI significantly, with a difference of 0⋅8 units between fish fed the diet containing the 16 and the diet containing the 25 % of dietary lipid inclusion at 8 °C, as well as at 15 °C. The effect of temperature was significant and averaged 0⋅5 units. The analyses of fatty acid composition of the PI showed that lipid accumulation was the main cause of the increase in OSIPI, increasing in sum of fatty acids by 8–9 %, independent of temperature (Fig. 2). The histological examination of the PI tissue (Fig. 3) gave results in line with observations of lipid content and showed severe increase in accumulation of lipid droplets in the cytosol of the enterocytes, i.e. steatosis, with increasing dietary lipid level at both temperatures (*P*=0⋅0003). The difference between the temperatures was not significant (*P*=0⋅3170).

**Table 2.** Results for all fish in the tanks regarding growth (SGR and TGC), and for sampled fish regarding body weights (BW, g), body length (BL, cm), yield, and organosomatic index of pyloric intestine (OSIPI, %), sum of fatty acids in pyloric caeca (LipPI, %), liver index (HSI, %), sum fatty acids in liver (LipLi, %) given as means of lipid level and temperatures, and treatment means, and statistics from two-way ANOVA

Temperature	Lipid	Performance, organ weights, lipid level								
		SGR	TGC	BW	BL	Yield	OSIPI	LipPI	HSI	LipLi
*Class means*										
	16	1⋅98	1⋅62	67⋅8	17⋅0	87⋅0^a^	3⋅3^b^	8⋅1^c^	1⋅2^b^	4⋅4^ab^
	20	1⋅92	1⋅62	70⋅7	17⋅2	87⋅0^a^	3⋅6^b^	13⋅9^b^	1⋅2^b^	5⋅0^a^
	25	1⋅90	1⋅54	66⋅4	17⋅0	86⋅5^b^	4⋅1^a^	20⋅2^a^	1⋅2^b^	4⋅8^a^
	28	1⋅96	1⋅57	68⋅3	17⋅1	86⋅4^b^	4⋅1^a^	16⋅6^ab^	1⋅4^a^	3⋅5^b^
8		1⋅14^b^	1⋅32^b^	47⋅2^b^	15⋅4^b^	86⋅2^b^	3⋅5^b^	14⋅8	1⋅5^a^	6⋅3^a^
15		2⋅74^a^	1⋅86^a^	89⋅4^a^	18⋅7^a^	87⋅2^a^	4⋅0^a^	14⋅4	1⋅1^b^	2⋅5^b^
*Tank means*										
8	16	1⋅21	1⋅39	46⋅2	15⋅3	86⋅7	3⋅0	8⋅8	1⋅4	6⋅2
8	20	1⋅19	1⋅31	44⋅9	15⋅2	86⋅4	3⋅5	13⋅4	1⋅4	7⋅4
8	25	1⋅10	1⋅29	49⋅9	15⋅6	85⋅8	3⋅8	19⋅0	1⋅5	6⋅7
8	28	1⋅05	1⋅27	47⋅9	15⋅5	85⋅9	3⋅7	17⋅8	1⋅6	5⋅0
15	16	2⋅74	1⋅85	89⋅4	18⋅7	87⋅3	3⋅5	7⋅3	1⋅0	2⋅6
15	20	2⋅65	1⋅94	96⋅6	19⋅2	87⋅6	3⋅7	14⋅4	1⋅0	2⋅5
15	25	2⋅70	1⋅79	82⋅9	18⋅4	87⋅1	4⋅3	21⋅4	1⋅0	2⋅9
15	28	2⋅87	1⋅87	88⋅8	18⋅7	86⋅9	4⋅5	15⋅3	1⋅2	2⋅0
Statistics (Two-way ANOVA)*										
*p*(model)		<0⋅0001	<0⋅0001	<0⋅0001	<0⋅0001	0⋅0003	0⋅0004	0⋅0004	<0⋅0001	<0⋅0001
*p*(temp)		<0⋅0001	<0⋅0001	<0⋅0001	<0⋅0001	<0⋅0001	0⋅0016	0⋅8638	<0⋅0001	<0⋅0001
*p*(lipid)		0⋅3285	0⋅4254	0⋅7969	0⋅9133	0⋅0248	0⋅0012	0⋅0002	0⋅0031	0⋅0892
Pooled sem		0⋅051	0⋅057	4⋅4	0⋅32	0⋅22	0⋅17	1⋅7	0⋅048	0⋅44

* The results are based on a model without interaction as a model with interaction gave insignificant results for the interactions. Yield = 100*(gutted weight/body weight).

Concerning HSI, no significant differences were observed between fish fed the 16 and 25 % lipid diets, but in the case of fish fed the diet with 28 % of lipid level, elevated the index significantly by 0⋅2 units at both temperatures, and an average difference between the temperatures of 0⋅4, lowest at the low temperature (Fig. 2).

### Growth performances and apparent nutrient digestibility

The fish grew well and only two fish, from different treatments, died during the feeding period. Feeding activity and consequently growth performance (Table 2) was significantly and positively affected by water temperature. Dietary lipid level did not affect final weight significantly. However, yield decreased with increasing lipid level from 16 to 25 %, whereas no further decrease was observed for the highest lipid level.

The results regarding nutrient digestibility are shown in Table 3. Lipid digestibility decreased slightly but significantly with increasing lipid level in the diet, whereas digestibility of CP, energy, ash and DM showed the opposite picture. Faecal DM was lower for the high lipid diet than for the other diets. Regarding effects of temperature, the only significant difference was observed for DM, for which digestibility was lower in fish raised at 15 compared to 8 °C.

**Table 3.** Results regarding apparent nutrient digestibility and faecal dry matter given as means of lipid level and temperatures, and treatment means, and statistics from two-way ANOVA

Temperature	Lipid level	Digestibility					
		Lipid	Protein	Ash	Energy	Dry matter	Faeces DM
*Class means*							
	16	95⋅8^a^	90⋅0^c^	34⋅6^b^		75⋅1^b^	12⋅5^a^
	20	96⋅2^a^	90⋅9^b^	37⋅6^ab^		79⋅0^b^	12⋅3^a^
	25	94⋅6^b^	91⋅5^a^	41⋅1^b^		79⋅0^b^	11⋅9^a^
	28	94⋅4^b^	91⋅8^a^	50⋅6^a^		83⋅0^a^	10⋅9^b^
8		95⋅2	90⋅9	42⋅8^a^		80⋅0^a^	12⋅0
15		95⋅2	91⋅2	39⋅2^b^	85⋅8	79⋅2^b^	11⋅8
*Tank means*							
8	16	95⋅8	90⋅0	37⋅0		76⋅0	12⋅7
8	20	96⋅3	90⋅8	40⋅3		79⋅6	12⋅6
8	25	94⋅5	91⋅3	42⋅7		81⋅4	12⋅2
8	28	94⋅4	91⋅4	51⋅1		83⋅1	10⋅7
15	16	95⋅8	90⋅0	32⋅2	81⋅9^c^	74⋅2	12⋅4
15	20	96⋅0	90⋅9	34⋅8	86⋅1^b^	78⋅4	12⋅0
15	25	94⋅8	91⋅7	39⋅6	87⋅1^ab^	81⋅2	11⋅6
15	28	94⋅3	92⋅2	50⋅1	88⋅1^a^	83⋅0	11⋅1
Statistics (Two-way ANOVA)*							
*p*(model)		0⋅0046	0⋅0002	<0⋅0001	0⋅0006	<0⋅0001	0⋅0028
*p*(temp)		0⋅9892	0⋅1005	0⋅0011		0⋅0414	0⋅2827
*p*(lipid)		0⋅0023	0⋅0001	<0⋅0001	0⋅0006	<0⋅0001	0⋅0016

* The results are based on a two-way model without interaction as a model with interaction gave insignificant results for the interactions. For digestibility of energy, faecal samples from fish fed at low temperature was insufficient and prevented analyses for energy content. For DE, a one-way analysis was performed, as sample size from the fish raised at low temperature was insufficient for analyses.

### Lipid content in pyloric and liver tissue

**Figure 4.**
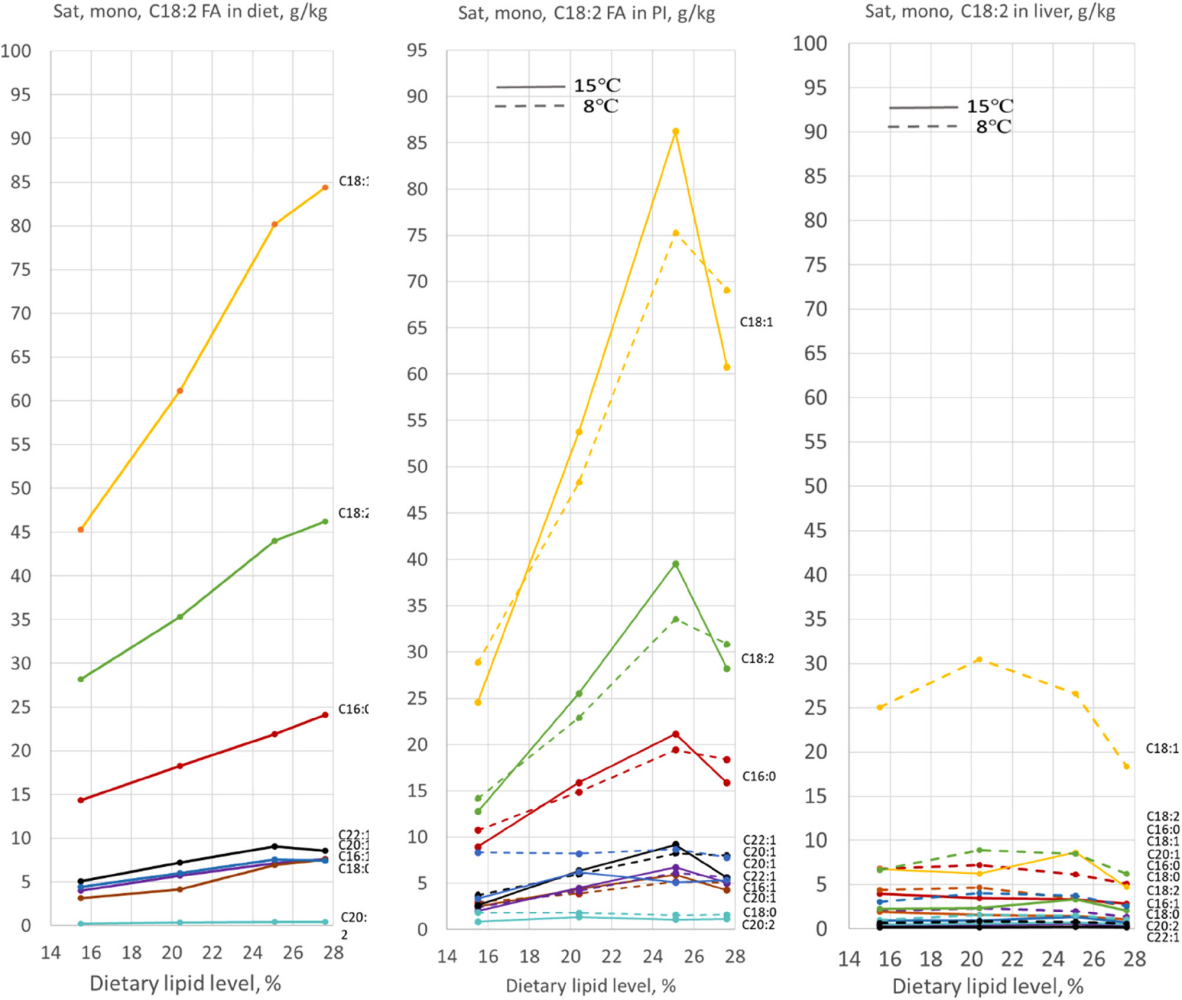
Level of saturated (Sat) and monounsaturated (MUFA) fatty acids in the diet (left), and in tissue from pyloric intestine (middle, PI) and liver (right), expressed as g per kg. The results of two-way ANOVA are shown in Tables 5 and 6.

Tables 1b, 4 and 5 present results regarding content of individual fatty acids in the diets, and in the tissues of the pyloric caeca and liver, expressed as relative values, i.e. % of total fatty acid. The corresponding results, in absolute amounts, i.e. expressed as g/kg tissue, are illustrated in Fig. 4. The statistical evaluations of these results are presented in Table 6 for the relative values, and in Table 7 for the absolute amounts. The results for the proportion of saturated, monounsaturated and C18:2 fatty acids (Tables 4–6) show that the profile for these fatty acids in the pyloric tissue, to a great extent, mirrored the diet, while the effects of temperature were minor. The liver showed a somewhat different picture, less clearly mirroring the diet composition, e.g. with lower level of C18:2, and with greater effects of temperature which differed in direction depending on the fatty acid in focus, e.g. regarding C16:0 and C18:1, both showing significant temperature effect. The content of *n*3 fatty acids in the pyloric tissue showed a more dynamic picture. Temperature effects were minor also for these fatty acids. The liver, again, showed a different picture with elevated levels of C22:6*n*3, and great, significant decreasing effect of increasing temperature.

**Table 4.** Content of saturated, monounsaturated, *n*6, *n*9 and *n*11 fatty acids, % of total fatty acids, in pyloric caeca and liver tissue with indicators of significance of effects of lipid level and temperature given as means of lipid level and temperatures, and treatment means*

Temperature	Lipid	C14:0	C16:0	C16:1*n*7	C18:0	C18:1*n*9c	C18:2*n*6c	C20:0	C20:1*n*11	C20:2*n*6	C20:3*n*6	C20:4*n*6	C22:1*n*11
*Pyloric caeca*													
	15⋅5	2⋅01^b^	10⋅73^a^	2⋅41^c^	2⋅97^a^	28⋅79^c^	14⋅70^b^	0⋅32	3⋅62^b^	0⋅94^a^	0⋅79^a^	0⋅50^a^	3⋅31
	20⋅4	2⋅10^ab^	9⋅72^a^	2⋅71^b^	2⋅61^b^	31⋅90^b^	15⋅19^b^	0⋅36	3⋅99^a^	0⋅87^b^	0⋅54^b^	0⋅33^b^	3⋅86
	25⋅1	2⋅14^a^	9⋅07^c^	2⋅86^ab^	2⋅47^c^	35⋅91^a^	16⋅26^a^	0⋅37	4⋅16^a^	0⋅84^bc^	0⋅43^c^	0⋅24^c^	3⋅86
	27⋅6	2⋅16^a^	9⋅43^bc^	2⋅91^a^	2⋅55^bc^	35⋅10^a^	16⋅07^a^	0⋅35	3⋅90^ab^	0⋅80^c^	0⋅41^c^	0⋅30^b^	3⋅51
8		2⋅09	9⋅60	2⋅68	2⋅59^b^	32⋅17^b^	14⋅93^b^	0⋅36	3⋅96	0⋅81^b^	0⋅55	0⋅36^a^	3⋅82
15		2⋅11	9⋅87	2⋅76	2⋅71^a^	33⋅68^a^	16⋅19^a^	0⋅34	3⋅88	0⋅92^a^	0⋅54	0⋅32^b^	3⋅46
8	15⋅5	2⋅03	10⋅46	2⋅38	2⋅88	27⋅99	13⋅82	0⋅34	3⋅80	0⋅90	0⋅80	0⋅52	3⋅59
8	20⋅4	2⋅07	9⋅58	2⋅67	2⋅50	31⋅00	14⋅76	0⋅35	3⋅91	0⋅84	0⋅55	0⋅36	3⋅84
8	25⋅1	2⋅18	9⋅16	2⋅87	2⋅43	35⋅37	15⋅78	0⋅36	4⋅14	0⋅78	0⋅45	0⋅26	3⋅87
8	27⋅6	2⋅11	9⋅21	2⋅81	2⋅53	34⋅34	15⋅35	0⋅38	3⋅98	0⋅74	0⋅39	0⋅30	3⋅96
15	15⋅5	1⋅99	10⋅99	2⋅44	3⋅05	29⋅60	15⋅57	0⋅31	3⋅44	0⋅98	0⋅77	0⋅48	3⋅03
15	20⋅4	2⋅14	9⋅87	2⋅75	2⋅72	32⋅80	15⋅63	0⋅37	4⋅08	0⋅91	0⋅53	0⋅29	3⋅88
15	25⋅1	2⋅10	8⋅99	2⋅84	2⋅51	36⋅46	16⋅75	0⋅37	4⋅17	0⋅91	0⋅42	0⋅21	3⋅86
15	27⋅6	2⋅21	9⋅64	3⋅00	2⋅57	35⋅86	16⋅80	0⋅32	3⋅83	0⋅86	0⋅42	0⋅30	3⋅06
*Liver*													
	16	1⋅26	11⋅61	2⋅14	6⋅28	28⋅20	8⋅30	0⋅16	3⋅55	1⋅55	2⋅23	1⋅63	0⋅76
	20	1⋅24	10⋅68	2⋅12	5⋅49	28⋅10	9⋅21	0⋅16	3⋅90	1⋅94	2⋅03	1⋅36	0⋅79
	25	1⋅24	9⋅43	2⋅05	4⋅29	28⋅64	10⋅20	0⋅16	4⋅27	2⋅10	1⋅66	1⋅22	0⋅90
	28	1⋅25	10⋅62	1⋅88	4⋅44	25⋅48	9⋅70	0⋅17	3⋅74	1⋅97	1⋅54	1⋅31	0⋅88
8		1⋅38	9⋅22	2⋅56	5⋅15	33⋅01	10⋅20	0⋅17	4⋅42	1⋅75	1⋅60	0⋅96	0⋅98
15		1⋅11	11⋅95	1⋅54	5⋅10	22⋅20	8⋅50	0⋅14	3⋅31	2⋅03	2⋅13	1⋅80	0⋅68
8	16	1⋅39	10⋅03	2⋅74	6⋅28	34⋅20	9⋅14	0⋅17	4⋅13	1⋅43	1⋅90	1⋅07	0⋅88
8	20	1⋅38	9⋅02	2⋅74	5⋅53	34⋅77	10⋅27	0⋅18	4⋅56	1⋅82	1⋅61	0⋅85	0⋅99
8	25	1⋅37	8⋅77	2⋅45	4⋅37	32⋅33	10⋅71	0⋅17	4⋅61	1⋅97	1⋅51	0⋅92	1⋅05
8	28	1⋅39	9⋅06	2⋅32	4⋅42	30⋅74	10⋅68	0⋅19	4⋅39	1⋅79	1⋅37	0⋅99	1⋅03
15	16	1⋅12	13⋅19	1⋅53	6⋅29	22⋅20	7⋅47	0⋅15	2⋅97	1⋅68	2⋅56	2⋅19	0⋅65
15	20	1⋅09	12⋅34	1⋅51	5⋅46	21⋅43	8⋅14	0⋅14	3⋅25	2⋅05	2⋅45	1⋅87	0⋅60
15	25	1⋅12	10⋅10	1⋅66	4⋅22	24⋅95	9⋅70	0⋅15	3⋅93	2⋅23	1⋅82	1⋅52	0⋅76
15	28	1⋅11	12⋅18	1⋅44	4⋅46	20⋅22	8⋅72	0⋅15	3⋅09	2⋅16	1⋅71	1⋅64	0⋅73

* *P*-values and pooled SEMs are given in Table 6.

**Table 5.** Sum of fatty acids (FA, % of diet) and content of *n*3 fatty acids (% of total FA) in pyloric caeca and liver tissue given as lipid level means, temperatures mean, and treatment means

Temperature	Diet lipid	C18:3*n*3	C20:5*n*3	C22:5*n*3	C22:6*n*3	Sum *n*3	Sum MUFA	Sum Sat
*In the pyloric caeca*								
	15⋅5	3⋅43^c^	1⋅80^a^	0⋅43^a^	8⋅95^a^	13⋅5	35⋅9	15⋅4
	20⋅4	3⋅66^b^	1⋅50^b^	0⋅34^b^	6⋅03^b^	18⋅4	68⋅6	24⋅7
	25⋅1	3⋅87^a^	1⋅26^c^	0⋅26^c^	4⋅15^c^	21⋅8	106⋅0	33⋅1
	27⋅6	3⋅94^a^	1⋅53^b^	0⋅31^b^	5⋅03^c^	19⋅8	85⋅2	27⋅8
8		3⋅67	1⋅68^a^	0⋅36^a^	6⋅26	19⋅6	73⋅9	25⋅6
15		3⋅79	1⋅36^b^	0⋅31^b^	5⋅82	17⋅0	73⋅2	24⋅8
8	15⋅5	3⋅32	1⋅93	0⋅46	9⋅01	15⋅2	39⋅4	16⋅8
8	20⋅4	3⋅60	1⋅73	0⋅37	6⋅45	19⋅2	65⋅2	23⋅8
8	25⋅1	3⋅80	1⋅43	0⋅29	4⋅52	21⋅8	99⋅4	31⋅6
8	27⋅6	3⋅94	1⋅64	0⋅33	5⋅04	22⋅3	91⋅7	30⋅1
15	15⋅5	3⋅54	1⋅67	0⋅41	8⋅88	11⋅8	32⋅4	14⋅0
15	20⋅4	3⋅72	1⋅28	0⋅32	5⋅61	17⋅6	72⋅0	25⋅6
15	25⋅1	3⋅94	1⋅08	0⋅23	3⋅77	21⋅8	113⋅2	34⋅8
15	27⋅6	3⋅95	1⋅41	0⋅29	5⋅02	17⋅4	78⋅7	25⋅6
*In the liver*								
	16	1⋅38	1⋅87	0⋅60	14⋅50	18⋅5	34⋅8	19⋅7
	20	1⋅65	1⋅97	0⋅53	15⋅12	19⋅5	35⋅1	18⋅0
	25	1⋅99	2⋅08	0⋅46	15⋅12	20⋅0	36⋅1	15⋅6
	28	1⋅97	2⋅40	0⋅57	17⋅62	22⋅9	32⋅2	17⋅0
8		1⋅93	1⋅62	0⋅38	10⋅53	14⋅7	41⋅2	16⋅4
15		1⋅56	2⋅53	0⋅70	20⋅65	25⋅7	27⋅9	18⋅8
8	16	1⋅55	1⋅39	0⋅39	9⋅35	12⋅8	42⋅1	18⋅3
8	20	1⋅88	1⋅43	0⋅35	9⋅03	12⋅9	43⋅2	16⋅5
8	25	2⋅11	1⋅78	0⋅37	11⋅48	16⋅0	40⋅6	15⋅1
8	28	2⋅19	1⋅90	0⋅41	12⋅26	17⋅0	38⋅7	15⋅5
15	16	1⋅21	2⋅35	0⋅81	19⋅65	24⋅2	27⋅5	21⋅2
15	20	1⋅42	2⋅52	0⋅72	21⋅21	26⋅1	27⋅0	19⋅5
15	25	1⋅88	2⋅38	0⋅55	18⋅77	23⋅9	31⋅5	16⋅0
15	28	1⋅75	2⋅89	0⋅74	22⋅98	28⋅7	25⋅7	18⋅4

**Table 6.** Results of two-way NOVA for content of fatty acids in tissue from the pyloric intestine (PI) and liver given as % of total fatty acids*

*In the pyloric intestine*							
	**C16:0**	**C16:1**	**C18:0**	**C18:1**	**C18:2**	**C20:1**	**C22:1**
*p*(model)	0⋅0002	0⋅0002	<0⋅0001	<0⋅0001	0⋅0009	0⋅0472	0⋅1834
*p*(temp)	0⋅1307	0⋅1744	0⋅0067	0⋅0019	0⋅0008	0⋅4886	0⋅1236
*p*(lipid)	0⋅0001	0⋅0001	<0⋅0001	<0⋅0001	0⋅0064	0⋅0289	0⋅2487
	**C18:3*n*3**	C20:5**	C22:5**	**C22:6**	Sum *n*3**	**Sum MUFA**	**Sum Sat**
*p*(model)	0⋅0003	0⋅0002	<0⋅0001	<0⋅0001	<0⋅0001	0⋅0082	0⋅0097
*p*(temp)	0⋅0549	0⋅0005	0⋅0009	0⋅1711	0⋅0647	0⋅9300	0⋅8628
*p*(lipid)	0⋅0002	0⋅0011	<0⋅0001	<0⋅0001	<0⋅0001	0⋅0041	0⋅005
*In the liver*							
	**C16:0**	**C16:1**	**C18:0**	C18:1**	C18:2**	C20:1**	**C22:1**
*p*(model)	<0⋅0001	<0⋅0001	<0⋅0001	<0⋅0001	<0⋅0001	<0⋅0001	<0⋅0001
*p*(temp)	<0⋅0001	<0⋅0001	<0⋅0001	<0⋅0001	<0⋅0001	<0⋅0001	<0⋅0001
*p*(lipid)	0⋅0088	0⋅0673	0⋅0077	0⋅0519	0⋅0316	0⋅0297	0⋅0972
	**C18:3*n*3**	**C20:5**	**C22:5**	C22:6**	Sum *n*3**	Sum MUFA**	**Sum Sat**
*p*(model)	<0⋅0001	0⋅0002	0⋅0255	0⋅0191	<0⋅0001	<0⋅0001	0⋅0001
*p*(temp)	<0⋅0001	<0⋅0001	0⋅0117	0⋅007	<0⋅0001	<0⋅0001	0⋅0003
*p*(lipid)	0⋅0156	0⋅1142	0⋅1027	0⋅1074	0⋅0107	0⋅0756	0⋅0005

* The results are based on a two-way NOVA model without interaction as a model with interaction gave insignificant results for the interactions. For the pyloric intestine the analyses were conducted on Log_10_-transformed data.

** The statistical evaluation showed significantly lower values for the highest lipid level (28 %) compared to the second highest (25 %).

Expressed as amount in the tissue (%, Tables 4, 5, and 7), the results show the same pattern in the pyloric caeca as well as in the liver, but with greater differences due to the pronounced differences in lipid content in the organs. The effect of the unintentional raise in choline level between diet containing 25 and 28 % lipid was particularly pronounced, with decrease in fatty acid accumulation in the pyloric caeca tissue for most of the fatty acids, an effect which was not seen in the liver.

**Figure 5.**
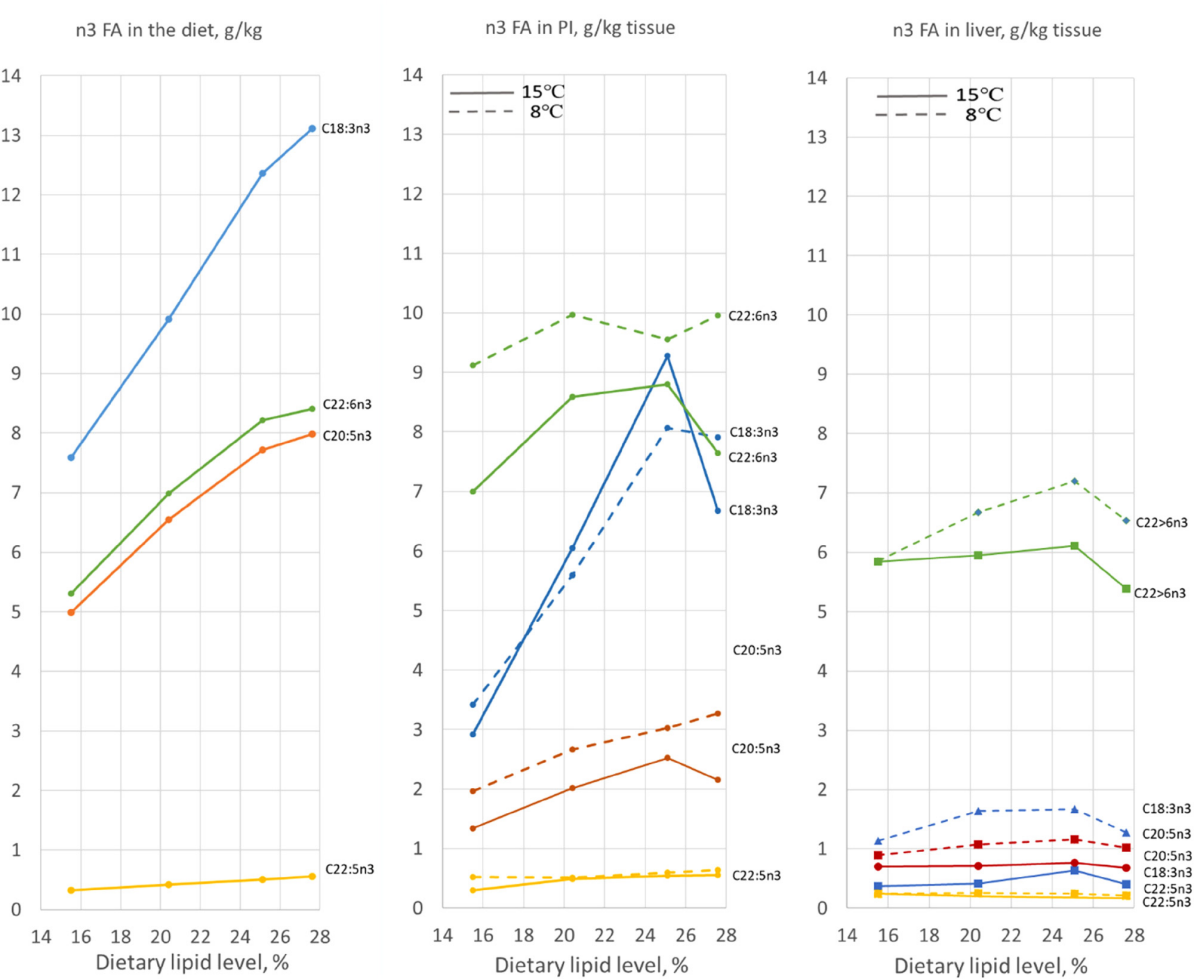
Level of *n*3 fatty acids in the diet (left), and in tissue from pyloric intestine (middle, PI) and liver (right), expressed as g per kg. The results of two-way ANOVA are shown in Tables 5 and 6.

The results for the *n*3 fatty acids (Fig. 5) appeared more dynamic, but with minor effect of temperature. The results for the liver indicate conversion of the C18:3*n*3 and C20:5*n*3 fatty acids to C22:6*n*3 this, and with clear temperature effects, in particular for C22:6*n*3. Table 7, which shows the quantitative aspects of these results, confirms the former results. They also illustrate the magnitude of the change in lipid metabolism, which was greater than indicated by the graphs which illustrate the results in g/kg tissue, whereas the tissue weight increased about 25 %. The drop in fatty acid level in the pyloric caeca between the diets containing 20 and 25 % of lipid level, most likely, was due to the higher choline content of this diet.

**Table 7.** Results of two-way NOVA for content of fatty acids in pyloric and liver tissue given as g/kg tissue

*In the pyloric intestine*							
	C16:0**	C16:1**	C18:0**	C18:1**	C18:2**	**C20:1**	**C22:1**
*p*(model)*	0⋅0008	<0⋅0001	0⋅0041	0⋅0001	0⋅0002	0⋅0465	0⋅0041
*p*(temp)	0⋅7200	0⋅9194	0⋅9917	0⋅8601	0⋅6168	0⋅5046	0⋅4612
*p*(lipid)	0⋅0004	<0⋅0001	0⋅002	<0⋅0001	<0⋅0001	0⋅0282	0⋅0022
	C18:3**	**C20:5**	**C22:5**	**C22:6**	Sum *n*3**	Sum MUFA**	Sum Sat**
*p*(model)*	0⋅0005	<0⋅0001	<0⋅0001	0⋅0464	<0⋅0001	<0⋅0001	<0⋅0001
*p*(temp)	0⋅9666	0⋅0002	0⋅0014	0⋅0081	0⋅0004	0⋅9987	0⋅5768
*p*(lipid)	0⋅0002	0⋅0003	<0⋅0001	0⋅3751	<0⋅0001	<0⋅0001	<0⋅0001
*In the liver*							
	**C16:0**	**C16:1**	**C18:0**	C18:1**	C18:2**	C20:1**	**C22:1**
*p*(model)*	0⋅0001	<0⋅0001	<0⋅0001	<0⋅0001	<0⋅0001	<0⋅0001	<0⋅0001
*p*(temp)	<0⋅0001	<0⋅0001	0⋅7768	<0⋅0001	<0⋅0001	<0⋅0001	<0⋅0001
*p*(lipid)	0⋅0166	0⋅0986	<0⋅0001	0⋅0637	0⋅0008	0⋅0039	0⋅0342
	C18:3*n*3**	**C20:5**	**C22:5**	**C22:6**	Sum *n*3**	Sum MUFA**	**Sum Sat**
*p*(model)*	<0⋅0001	<0⋅0001	0⋅0001	<0⋅0001	<0⋅0001	<0⋅0001	<0⋅0001
*p*(temp)	<0⋅0001	<0⋅0001	<0⋅0001	<0⋅0001	<0⋅0001	<0⋅0001	<0⋅0001
*p*(lipid)	<0⋅0001	0⋅0308	0⋅1547	0⋅039	0⋅0253	0⋅0513	0⋅0105

* The results are based on a model without interaction as a model with interaction gave insignificant results for the interactions.

** The statistical evaluation showed significantly different values for the diet with the highest lipid level (28 %) compared to that of the second highest level (25 %).

### Intestinal gene expression

**Figure 6.**
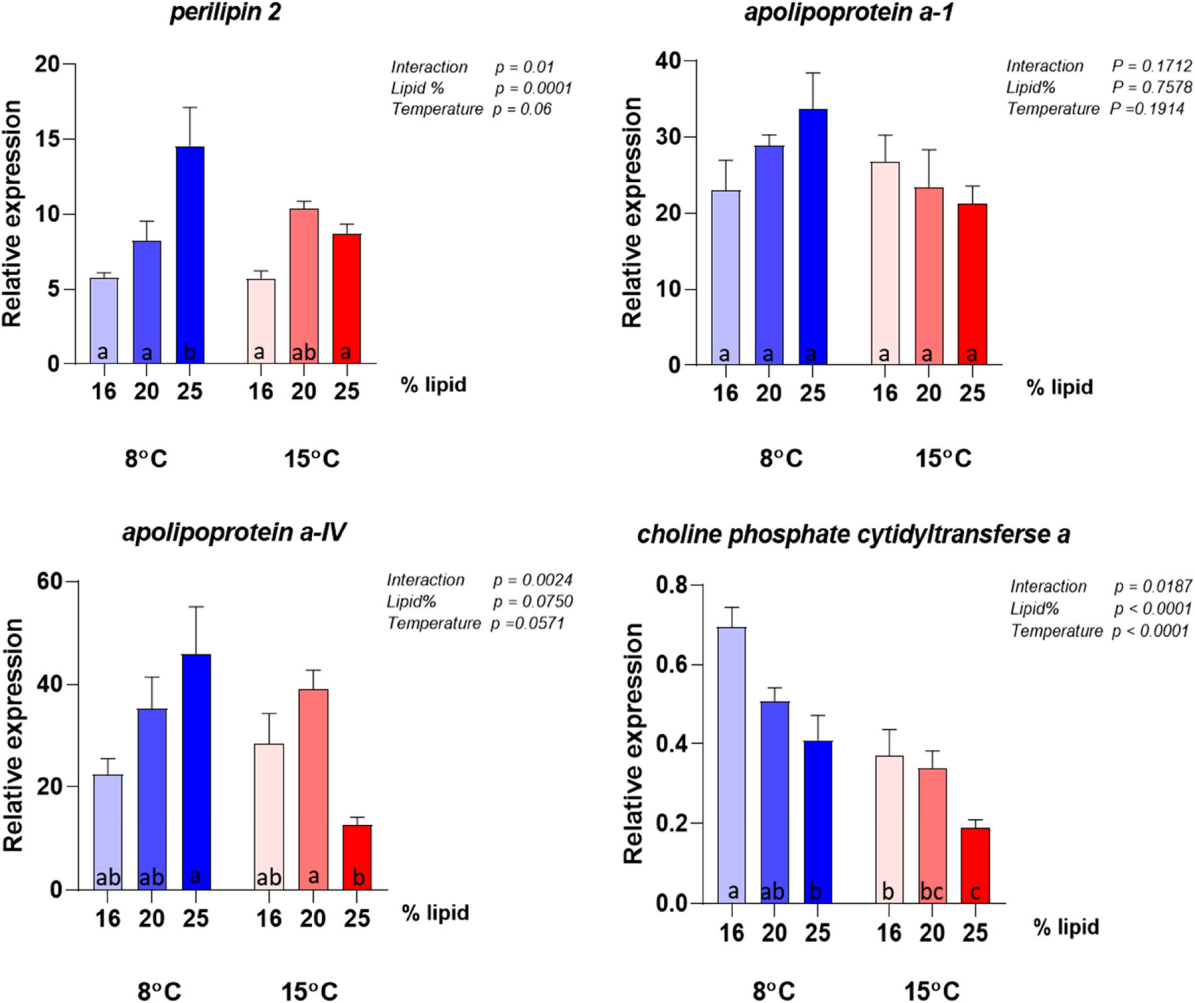
Expression of biomarker genes for choline requirement. Data are mean normalised expression levels + sem. Different letters denote statistically significant differences among diet groups.

**Figure 7.**
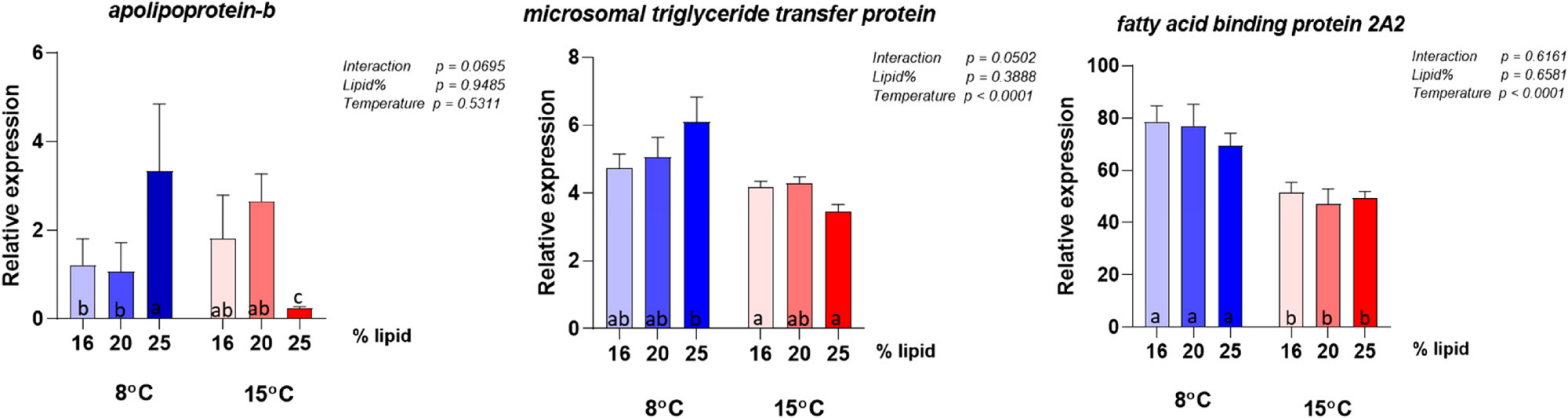
Expression of genes involved in lipid and fatty acid transport. Data are mean normalised expression levels + sem. Different letters denote statistically significant differences among diet groups.

**Figure 8.**
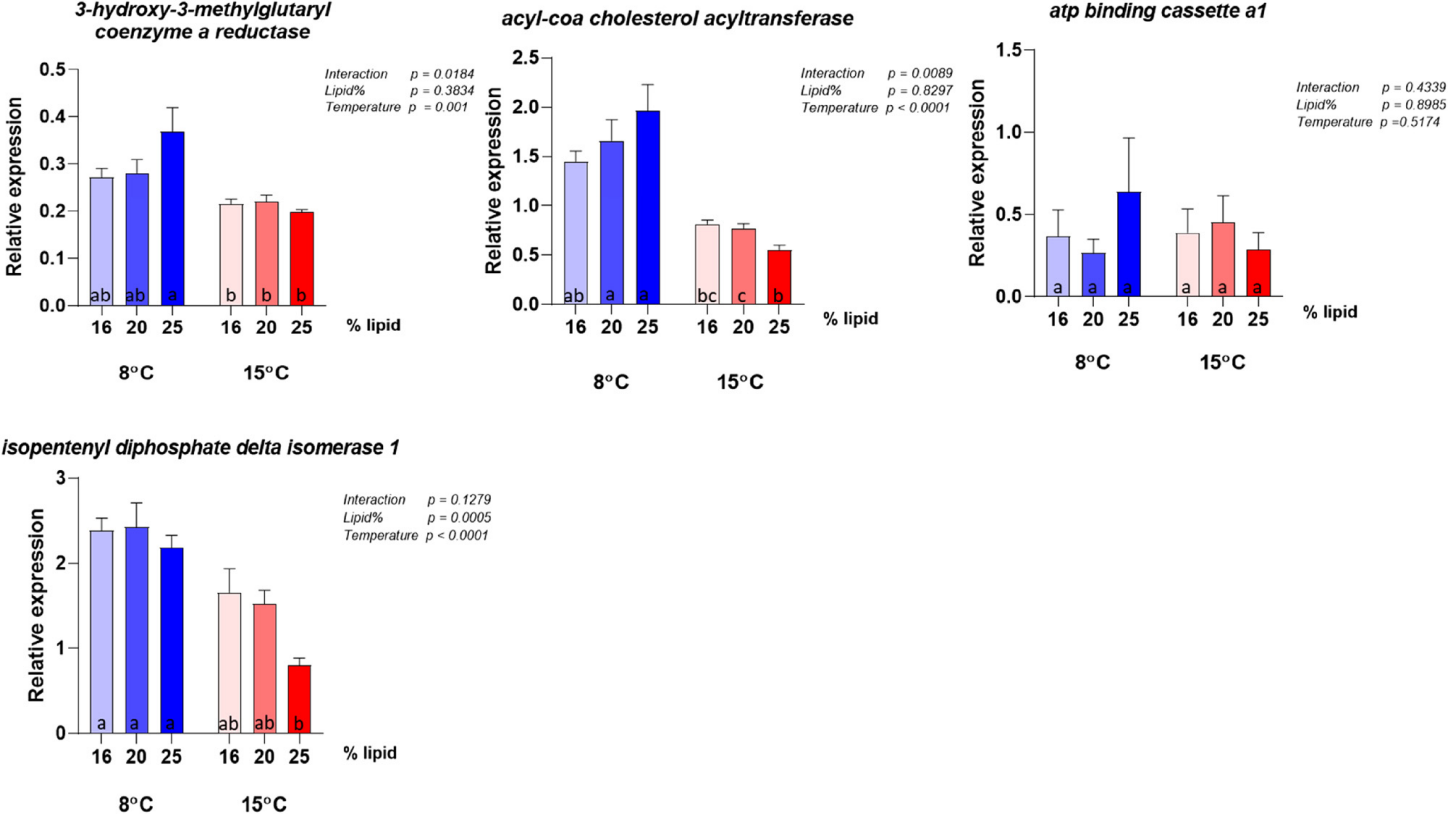
Expression of genes involved in cholesterol metabolism. Data are mean normalised expression levels + sem. Different letters denote statistically significant differences among diet groups.

**Figure 9.**
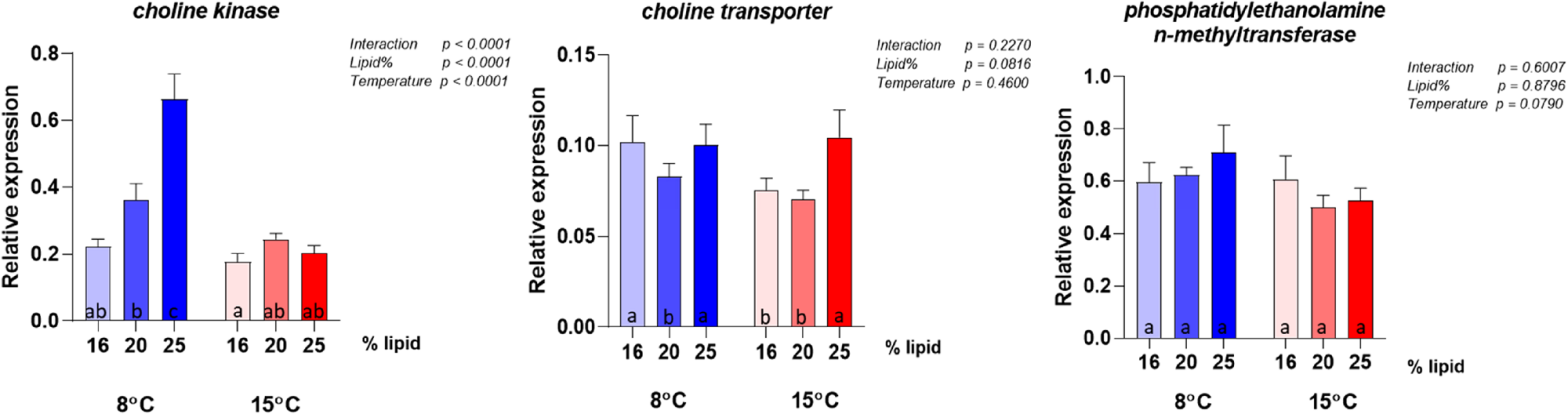
Expression of genes involved in phosphatidylcholine synthesis. Data are mean normalised expression levels + sem. Different letters denote statistically significant differences among diet groups.

**Figure 10.**
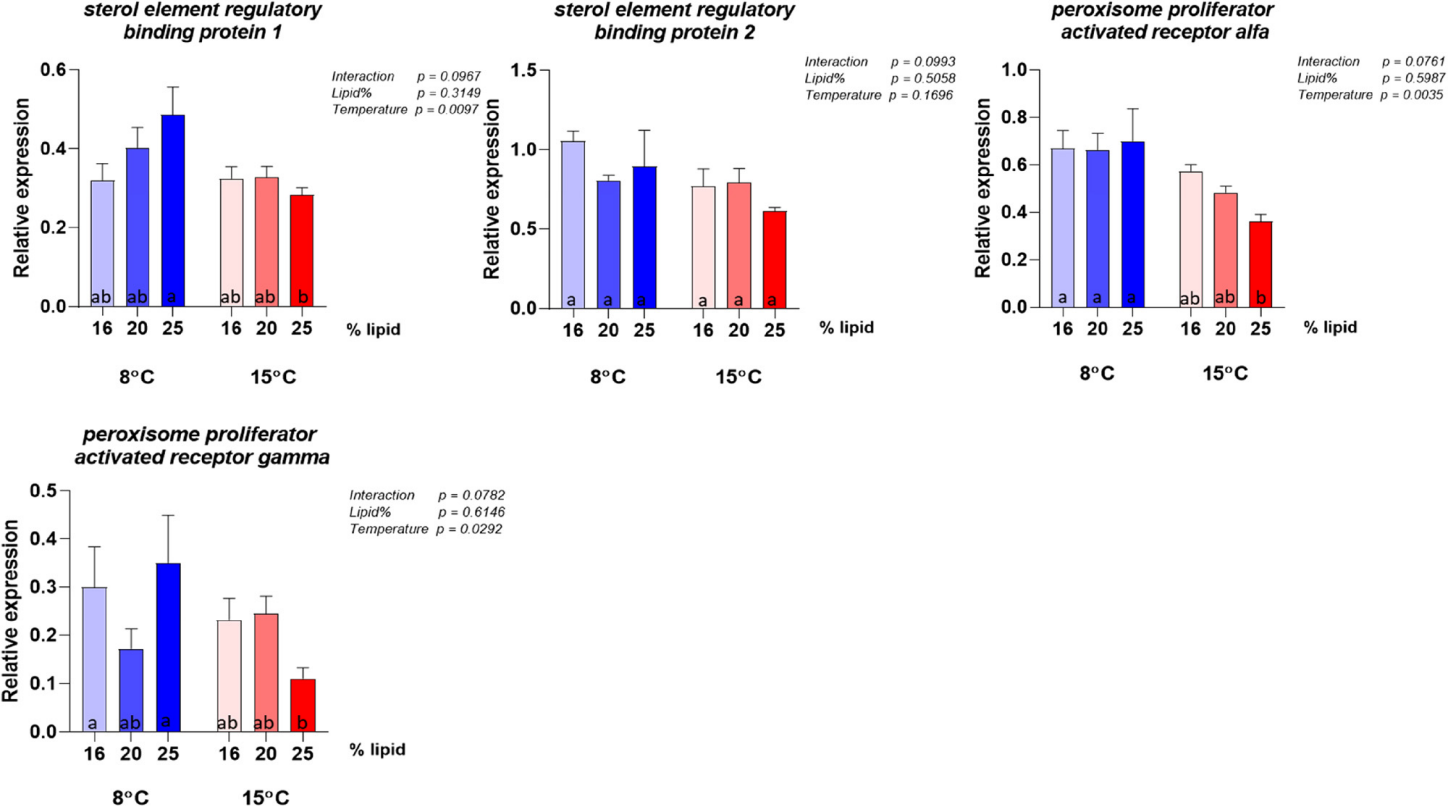
Expression of genes coding for nuclear receptors involved in regulation of lipid and sterol metabolism. Data are mean normalised expression levels + sem. Different letters denote statistically significant differences among diet groups.

The gene expression analyses were conducted on the same panel of genes selected by Hansen *et al.*^(10)^ to describe possible metabolic alterations induced by variation in choline supply and support discussion regarding choline requirement. The results for the highest lipid diet (28 %) did not give meaningful results in this context, due to the event in the feed production described above and are therefore not included in the interpretation of the molecular results. Selected genes showing clear effects of either dietary lipid level or temperature are illustrated in Fig. 6 and complete results are presented in Figs. 7–10. Overall, the expression of the genes were higher at 8 °C than at 15 °C, and there was a significant or close to significant interaction between temperature and dietary lipid level for all the genes. Whereas most of the genes at 8 °C showed increasing expression with increasing lipid level, the expression increased or was stable between the diet containing 16 and 20 % of fat and decreased, or tended to decrease, between the 20 and 25 % at 15 °C. The exception was *pcyt1α* which showed decreasing expression with increasing lipid level at both temperatures (Fig. 6). At 8 °C, the expression of *plin2* was significantly upregulated with increasing lipid level, while at 15 °C, the pattern was slightly different showing an upregulation up to 20 % of fat inclusion, for then to decrease at 25 % of dietary lipid level. Concerning both *apoA-I* and *apoA-IV*, at 8 °C their expression followed the same dose-response curve observed for *plin2*. However, at 15 °C, the picture was less clear. While *apoA-IV* showed the same pattern observed in *plin2*, with the expression peaking at 20 % dietary lipid and then decreasing with the highest lipid percentage, *apoA-I* showed an inverse relationship, being highly expressed with the lowest lipid inclusion. The curve describing the expression of *pcyt1α* followed at both temperatures a clear inverse relationship with the dietary lipid inclusion. The same trend was observed at 15 °C. The majority remainder of the assessed (Figs. 7–10) genes showed the same expression pattern already observed in the biomarker genes (Fig. 6). At 8 °C, the genes involved in fatty acid synthesis, like *srebp1*, *acat* and *hmgcr*, were upregulated with the increasing lipid level. On the other hand, the picture observed at 15 °C was less clear and no significant difference could be observed between the three different lipid levels. Concerning *srebp2*, another important gene involved in fatty acid synthesis, its expression showed a completely different picture, being only slightly influenced by water temperature and not by lipid level. Among the genes dedicated to the lipid transport, at 8 °C *mtp* showed the same dose-response effect already observed in other genes. Concerning the enzymes participating in phosphatidylcholine synthesis, the expression of *chk* was significantly enhanced by increasing lipid levels by the lower temperature and the higher lipid level, while it was downregulated at 15 °C and there was no difference between the three lipid levels.

## Discussion

### Effects on organosomatic index and cell vacuolation of the pyloric caeca

The results show that among the biomarkers selected for evaluation of state of choline supply, the most sensitive indicators were related to the pyloric ceaca, i.e. OSIPI, histological observation of cell vacuolisation and lipid accumulation, in line with the results showed by Hansen *et al.*^(10)^. All the indicators increased with increasing dietary lipid content, and partly with increased temperature, probably as a consequence of the higher feed intake given by the higher water temperature. The temperature effects observed in the present study are parallel to results observed in in rainbow trout by Ng *et al.*^(36)^. The mechanisms underlying these observations were, most likely, related to choline's role in lipid transport. Choline deficiency leads to insufficient transport of fat from the cytosol across the basolateral cell membrane of intestinal enterocytes. As discussed below, the cell metabolism is consequently altered towards temporary storage of the lipid in intracellular lipid vacuoles, resulting in excessive lipid accumulation^(37–40)^.

### Effects on growth performance and lipid digestibility

The results regarding effects of environmental temperature on growth performance are in accordance with findings of several other studies conducted on Atlantic salmon^(41–43)^ and other fish species such as rainbow trout^(44)^, darkbarbel catfish (*Pelteobagrus vachellii*)^(45)^ and yellowtail kingfish (*Seriola lalandi*)^(46)^. As fish are poikilotherms, the observation that fish raised at 15 °C grew 40 % more than those raised at 8 °C was as expected. The overall mechanism underlying this effect is the impact of the higher temperature on the metabolism of the fish, which leads to a concomitant increase in feed intake and growth^(47,48)^. The marginal effects of variation in temperature on lipid digestibility are partly in line with the results of a study of Grisdale-Helland *et al.*^(49)^. Their work showed that with soybean oil as source of lipid, temperature, 5 *v.* 12 °C, did not affect lipid digestibility significantly. However, when fish oil was the source, lipid digestibility increased with temperature. The conclusion was that lipids with low melting point, such as plant oils, are highly digestible and are only slightly influenced by the environmental temperature. On the other hand, for lipids with high melting point, which means high level of saturated and/or long-chain polyunsaturated fatty acids, overall digestibility is lower and is affected to a larger extent by temperature^(36,50,51)^.

The observed increase in digestibility of protein and energy with increasing lipid level, and to a more pronounced degree for ash digestibility, was supposedly related to an increase in gut passage time induced by the increase in lipid level, allowing more time for digestion and absorption. This is a well-known relationship in animals^(52–54)^.

### Effects on lipid content in the pyloric caeca and liver

The results observed for fatty acid composition in the diet, pyloric caeca and liver, illustrate that the pyloric caeca markedly modulate the fatty acid profile of the diet and the liver. This regards in particular the *n*3 fatty acids, for which there seemed to be a substantial conversion from 18:3*n*3 and 20:5*n*3 to 22:5*n*3. This observation is in line with the results of Bou *et al.*^(5)^, which showed a clear difference between the fatty acid composition of the diet and that of tissue from the MI. In the present work, the conversion of the *n*3 fatty acids seemed to be affected the most by lipid level of the diet. This observation was possibly a consequence of the increasing severity of the steatosis which developed in the enterocytes. The abrupt change in the general development of this relationship between the diet containing 25 and 28 % of fat, most clearly illustrated for the results expressed as g/kg, supports this consideration. The marked difference in fatty acid composition between the liver and the pyloric caeca, and the greater, and seemingly opposite effect of temperature, more marked and inverse for the liver, underlines the different roles of these two organs. These results also call for better understanding of the pathways and metabolism of lipid between organs in fish. Knowledge on the transport routes from the intestine to the peripheral tissues and internal organs is weak. Although a lymphatic system has been described in fish, with zebrafish as the main model^(55)^, an intestinal lymphatic system has not been identified in Atlantic salmon. As there are species differences among vertebrates regarding lymph vessels in the intestine, e.g. a lymphatic system is absent in chicken^(56)^, efforts should be made to identify lipid transport routes for Atlantic salmon. Such information would help understand the trafficking and metabolism of lipid in this important domesticated animal.

### Effects on gene expression in the pyloric caeca

Among the assessed genes, *plin2*, *apoA-I*, *apoA-IV* and *pcyt1α* showed the greatest effects of lipid and temperature and are therefore considered to comprise the most sensitive biomarkers for choline requirement, in accordance with the observations in the study of Hansen *et al.*^(10)^ In Hansen *et al.*'s dose-response study, in which dietary lipid level was the same (29 %) for all groups, the expression of *plin2* followed an inverse relationship with choline and degree of enterocyte lipid accumulation. The inverse relationship between intestinal *plin2* expression and steatosis has been observed in several recent studies in Atlantic salmon^(9,11,57–59)^, and highlights that *plin2* is a sensitive biomarker of intestinal lipid accumulation^(60)^. In the present study, in which choline inclusion was kept equally low for all diets while the lipid level increased, *plin2* expression followed a clear dose-response relationship with lipid percentage at 8 °C, again showing an inverse relationship with the degree of steatosis. This result not only confirmed *plin2*'s important role as indicator of choline requirement, but it also confirmed that the requirement is influenced by dietary lipid level. At 15 °C, *plin2* followed the same pattern only for the first two lipid levels, while at 25 % the expression dropped. The cause of the difference in response at the low and high temperature is difficult to suggest based on the other results of this study, and no scientific literature is available offering further information. Concerning the two genes encoding respectively for ApoA-I and ApoA-IV, major protein components of lipoproteins, they showed at 8 °C the same picture observed for *plin2*. This response matches the results obtained in Hansen *et al.*'s latest work, in which *apoa-I* and *apoa-IV* were both suppressed by a too low choline supply, while their expression was higher when choline level was adequate to provide a proper assembly of lipoproteins and sufficient lipid transport. At 15 °C, the picture showed by the expression levels of the two genes, especially of *apoa-I*, again indicated that the result was affected by the environmental temperature, as confirmed by the significance of the interaction term. Among the genes involved in phosphatidylcholine production, *chk*, which regulates the first step of the cytidine (CDP)-choline pathway^(35,36)^ and phosphorylates choline, showed the same expression pattern already discussed for *plin2* at both 8 and 15 °C. On the contrary, *pcyt1a*, involved in the generation of the high energy donor CDP-choline by regulating the second step of the pathway^(44,46)^, showed a different picture, being strongly downregulated by the increasing lipid level at both water temperatures. The *chk* expression pattern was in agreement with results presented in Hansen's dose-response study^(61)^, which showed that to a higher choline inclusion corresponds a lower *chk* expression. On the other hand, the opposite response of *pcyt1a*'s contrasted with the Hansen *et al.*^(10)^'s findings which showed similar responses in *pcyt1a* compared to the response in *chk*. Following the steps of the cytidine (CDP)-choline pathway, we can suggest, as possible explanation, that to a higher choline phosphorylation, corresponds a lower production of the high-energy donor CDP-choline. However, the complexity of the pathway, regulated by several rate-limiting enzymes^(62)^, makes it difficult to discuss the effects of the diet on *pcyt1a* expression and more specific studies are therefore needed.

At 8 °C, the expression of other genes involved in lipid metabolism, such as *hmgcr*, the rate-limiting enzyme for cholesterol synthesis, *acat* which catalyses cholesteryl esters synthesis from cholesterol^(63)^, the two transcription factors *srebp1* and *srebp2*, and *mtp*, which has a pivotal role in lipoprotein formation, confirmed the direct relationship between dietary lipid level and choline requirement. At 15 °C, all these genes followed the same pattern observed for *plin2*, validating the hypothesis of an interaction between water temperature and lipid inclusion. As the steatosis, as well as the lipid accumulation in the pyloric tissue indicated increased choline requirement with increasing temperature, it is unlikely that the gene expression results observed for the fish fed at high temperature, indicated mitigating effects.

No impact of the increasing fat level was observed for the liver, neither as indicated by the HSI or histology. This outcome is in accordance with the findings achieved in our previous studies^(9,10)^ which showed that liver lipid indicators are not suitable biomarkers for estimation of severity of lipid accumulation and, hence, for choline requirement^(64)^.

### Effects of lipid level and environmental temperature on choline requirement

According to the discussion above, the design of our experiment appears to be suitable for evaluation of possible effects of variation in dietary lipid content and temperature on choline requirement. To get an indication of the quantitative effects on choline requirement, the dose-response study conducted by Hansen *et al.*^(10)^'s work to estimate choline requirement in Atlantic salmon^(10)^ can be used, although the fish was somewhat larger and kept in seawater. In Hansen *et al.*'s^(10)^ work, a diet with a choline level of about 1900 mg/kg gave fish with an OSIPI of 2⋅0, while a diet with 1200 mg/kg resulted in a OSIPI of 2⋅4. Based on this observation, the results obtained from the present study provide an estimation of the effects on choline requirement given by the raise in lipid level from 16 to 25 %. With a dietary choline level of 1400 mg/kg, the shift of lipid level increased OSIPI by 0⋅8, regardless of the temperature. The effect of temperature, i.e. of an increase in OSIPI of 0⋅5, correspond to an estimated increase in choline requirement of about 900 mg/kg. As the conditions of the present and Hansen *et al.*'s study differ somewhat, the estimation of the effects of dietary lipid content and temperature, are high, indicating that lipid level in the diet, as well as temperature should be taken into account when diets are formulated. To be able to conclude the optimal level of choline in salmon diets for elimination of steatosis, a dose-response trial is needed with diets high in lipid and at high temperatures. Such an experiment should also observe endpoints regarding disease resistance as well, as choline is the primary source of methyl groups in epigenetic processes, which are essential for differentiation of immune cells.

## Conclusions

The obtained results confirmed the relevance of dietary lipid level and water temperature as important drivers for intestinal lipid accumulation, showing clear effects on steatosis symptoms on a molecular, histological, biochemical level. In addition, the effects given by the water temperature seemed particularly significant when the interaction with the different lipid levels occurs. These findings represent the first steps towards the estimation of choline requirement in Atlantic salmon at later developmental stages and raised under different environmental conditions. However, further analyses and a dose-response feeding trial will be needed to investigate how this interaction occurs and influences the mechanisms and the pathways behind lipid metabolism and choline requirement.

## Supplementary material

For supplementary material accompanying this paper visit https://doi.org/10.1017/jns.2023.45. click here to view supplementary material
