# Supplementary material for: Effects of dietary lipid level and environmental temperature on lipid metabolism in the intestine and liver, and choline requirement in Atlantic salmon (Salmo salar L) parr
Source: J Nutr Sci. 2023 May 25;12:e61. doi: 10.1017/jns.2023.45 (PMC10214143; doi:10.1017/jns.2023.45)
Supplement: Supplementary file 1 [file S2048679023000459sup001.docx]

Supplementary Table 1. Primer pair sequences, efficiency, amplicon size and annealing temperature for the genes used for real-time PCR.

| **Gene name** | **Gene symbol** | **Forward primer** | **Reverse primer** | **Amplicon**  **size** | **Annealing**  **temperature** | **Efficiency** | **Acc. number** |
| --- | --- | --- | --- | --- | --- | --- | --- |
| 3-hydroxy-methylglutaryl-coenzyme A reductase | *hmgcr* | CCTTCAGCCATGAACTGGAT | TCCTGTCCACAGGCAATGTA | 224 | 60 | 1.9 | NM_001173919 |
| Acyl-coA cholesterol acyltransferase | *acat* | TGCTGGAGTTTGACCTGTTG | GCTGCGATGGTAGAGAGTCC | 139 | 60 | 2.0 | GE793368 |
| Perilipin2 | *pli2* | CCCAGGTCTACTCCAGCTTC | CAGCGACTCCTTCATCTTGC | 104 | 60 | 2.0 | BT072598 |
| Apolipoprotein A-I | *apoA-I* | CTGGTCCTCGCACTAACCAT | TGGACCTCTGTGCAGTCAAC | 144 | 60 | 2.0 | NM_001123663 |
| Apolipoprotein A-IV | *apoA-IV* | CAGGACCAGTCTCAGCAACA | GTTGACTTCCTGTGCCACCT | 131 | 60 | 1.9 | BT048822 |
| Apolipoprotein B | *apo-B* | CCCTGAGATGGTGTCCGTAT | GCGTCGACTTCCATAGCTTC | 131 | 63 | 1.9 | CB504205 |
| ATP-binding cassette A1 | *abca1* | ACAGTGGAGGGAACATGAGG | CCCCTCCTTGACGATACTGA | 149 | 60 | 2.0 | TC187143 |
| Choline Kinase | *chk* | CTCAAGTTTGCCCGTCTGAT | CACAGGGGAATGAGTGGAGT | 88 | 60 | 1.9 | DY706802 |
| Choline transporter | *slc44a2* | TCGTCATCATTTTGCTGCTC | AGGCGATGACAATGGATAGG | 152 | 60 | 2.0 | NM_001140367 |
| Choline-phosphate cytidylyltransferase | *pcyt1a* | CGGGTCTATGCAGATGGAAT | GCTCGTCCTCGTTCATCACT | 166 | 60 | 2.1 | BT045986 |
| Cytochrome P450 51 | *cyp51* | TGCATTGGGGAGAACTTTGC | ATCTGATGACGGGGTTGTGT | 148 | 60 | 1.9 | XM_014177708 |
| Fatty acid binding protein 2b | *fabp2b* | TGCCTTCCCCTCATTCTCTA | GGTGATACGGTCTTCATCCAA | 82 | 60 | 2.0 | BT046827 |
| Fatty acid transport protein | *fatp* | AGGAGAGAACGTCTCCACCA | CGCATCACAGTCAAATGTCC | 159 | 60 | 1.9 | CA373015 |
| Isopentenyl-diphosphate delta isomerase 1 | *idi1* | TACCTCCCAAAATGGCACTC | CGTCCCTCATAGCAGCTTTC | 132 | 60 | 1.9 | XM_014157452 |
| Microsomal triglyceride transfer protein | *mtp* | AACGTGACAGTGGACATGGA | GGACCGTGGTGATGAAGTCT | 89 | 60 | 2.0 | CA042356 |
| Peroxisome proliferator activated receptor alpha | *pparα* | GCTTCATCACCAGGGAGTTT | TCACTGTCATCCAGCTCCAG | 113 | 60 | 2.0 | XM_036945541.1 |
| Peroxisome proliferator activated receptor gamma | *pparγ* | TGCTGCAGGCTGAGTTTATG | CAGGGGAAAGTGTCTGTGGT | 107 | 58 | 2.0 | XM_036984365.1 |
| Sterol element regulatory binding protein 1 | *srebp1* | GCCATGCGCAGGTTGTTTCTTCA | TCTGGCCAGGACGCATCTCACACT | 151 | 63 | 1.9 | HM561860 |
| Sterol element regulatory binding protein 2 | *srebp2* | TCGCGGCCTCCTGATGATT | AGGGCTAGGTGACTGTTCTGG | 147 | 60 | 1.9 | HM561861 |
| Phosphatidylethanolamine N-methyltransferase | *pemt* | GTTGCTGTCATCGCCATCAT | GAGGAGGATGATGAGGGTGC | 141 | 60 | 2.0 | BT049029 |
